# High-Density Lipoprotein Is Located Alongside Insulin in the Islets of Langerhans of Normal and Rodent Models of Diabetes

**DOI:** 10.3390/nu16020313

**Published:** 2024-01-20

**Authors:** Sahar Mohsin, Haba Elabadlah, Mariam K. Alotaiba, Suhail AlAmry, Shamma J. Almehairbi, Maha M. K. Harara, Aisha M. H. Almuhsin, Saeed Tariq, Frank Christopher Howarth, Ernest A. Adeghate

**Affiliations:** 1 Department of Anatomy, College of Medicine & Health Sciences, United Arab Emirates University, Al Ain P.O. Box 15551, United Arab Emirates; smohsin@uaeu.ac.ae (S.M.);; 2 Cambridge Medical and Rehabilitation Center, Al Ain P.O. Box 222297, United Arab Emirates; 3 Department of Physiology, College of Medicine & Health Sciences, United Arab Emirates University, Al Ain P.O. Box 15551, United Arab Emirates; chris.howarth@uaeu.ac.ae; 4 Zayed Centre for Health Sciences, United Arab Emirates University, Al Ain P.O. Box 15551, United Arab Emirates

**Keywords:** high-density lipoproteins, diabetes mellitus, pancreas, islet cells, immunofluorescence, electron microscopy, animal models of diabetes, high-fat diet

## Abstract

Recent studies have implicated pre-beta and beta lipoproteins (VLDL and LDL) in the etiopathogenesis of complications of diabetes mellitus (DM). In contrast, alpha lipoprotein (HDL) is protective of the beta cells of the pancreas. This study examined the distribution of HDL in the islets of Langerhans of murine models of type 1 diabetic rats (streptozotocin (STZ)-induced DM in Wistar rats) and type 2 models of DM rats (Goto–Kakizaki (GK), non-diabetic Zucker lean (ZL), and Zucker diabetic and fatty (ZDF)). The extent by which HDL co-localizes with insulin or glucagon in the islets of the pancreas was also investigated. Pancreatic tissues of Wistar non-diabetic, diabetic Wistar, GK, ZL, and ZDF rats were processed for immunohistochemistry. Pancreatic samples of GK rats fed with either a low-fat or a high-fat diet were prepared for transmission immune-electron microscopy (TIEM) to establish the cytoplasmic localization of HDL in islet cells. HDL was detected in the core and periphery of pancreatic islets of Wistar non-diabetic and diabetic, GK, ZL, and ZDF rats. The average total of islet cells immune positive for HDL was markedly (<0.05) reduced in GK and ZDF rats in comparison to Wistar controls. The number of islet cells containing HDL was also remarkably (*p* < 0.05) reduced in Wistar diabetic rats and GK models fed on high-fat food. The co-localization study using immunofluorescence and TIEM techniques showed that HDL is detected alongside insulin within the secretory granules of β-cells. HDL did not co-localize with glucagon. This observation implies that HDL may contribute to the metabolism of insulin.

## 1. Introduction

The continuous rise in the worldwide prevalence of diabetes mellitus (DM) is a cause of concern [1]. DM is triggered by a decreased release or impaired action of insulin, resulting in impaired metabolism of fat, protein, and carbohydrates. The abnormal metabolism of these molecules, especially that of carbohydrates, results in hyperglycemia. The diabetes-induced hyperglycemia causes the development of oxidative stress (OS). OS, in turn, enhances the generation of diabetes complications including neuropathy, angiopathy, nephropathy, and other chronic conditions [2]. The explosion in the total sum of people with diabetes correlates strongly with that of obesity [3]. This observation has prompted researchers to examine the role that lipids might play in the pathogenesis of this chronic disease. The association of DM with either subcutaneous or visceral obesity has been frequently referred to as “Diabesity”. Therefore, the significance of the interwoven effect of these two conditions is well appreciated in the literature. In obesity, pro-inflammatory cytokines, hormones, and other molecules that contribute to the initiation of insulin resistance (IR) are increased [3,4]. Abnormal lipid metabolism leading to the accumulation of triglycerides, beta lipoproteins (LDL) and pre-beta lipoproteins (VLDL) in blood circulation is a leading factor in triggering IR [5,6,7]. Further, their contribution to the etiopathogenesis of IR, elevated levels of fat components in the blood stream, can cause lipotoxicity on pancreatic beta cells, since beta cells are sensitive to abnormally high levels of lipid molecules [8]. The failure of islet of Langerhans beta cells will inevitably result in overt-type 2 DM (T2DM).

Data obtained from the UK (United Kingdom of Great Britain) Prospective Diabetes Study (UKPDS) involving a cohort of 2693 patients with a new onset T2DM showed a significant reduction in high-density lipoproteins (HDL) [9]. HDL is known to be responsible for removing “bad” cholesterol such as VLDL from the circulation back into the liver for conversion to non-harmful molecules [10]. Low-density lipoproteins are removed in the form of cholesterol esters [10]. The reduction in the level of HDL would imply that the body is unable to remove low-density lipoproteins (LDL) and VLDL from blood circulation. This UKPDS observation points to dyslipidemia, a complication of DM. Diabetes-induced dyslipidemia is also associated with increased levels of serum triglycerides, in addition to the high levels of VLDL and LDL particles [11].

It was shown that while VLDL induces apoptotic process in the islet of Langerhans, HDL was able to increase pancreatic beta cell mass [12]. Recent results show that incubation of pancreatic Min6 and primary islet cells with reconstituted HDL results in increased insulin release [13].

It was proposed that the capacity of HDL to improve beta cell physiological activity is attributed to its capability to protect beta cell from the dysfunction, cell stress, apoptosis and inflammatory insults caused by excess cholesterol perturbation [14]. Many systemic effects of HDL have been described. These include cardioprotection [15], which is achieved by its ability to clear macrophage-engulfed LDL (“bad” cholesterol) from the sub-endothelium [16,17]. In addition, HDL, as part of the lipid profile, is used as a biomarker for cardiovascular conditions and to monitor the effects of medications on the cardiovascular and metabolic parameters [18,19,20].

These observations appear, therefore, to have drawn the attention of researchers on the possible role of lipoproteins in the pathogenesis of DM. The present study examines whether HDL has any association with the endocrine pancreas function. If yes, what endocrine cell type is it associated with in both the normal and diabetic states? The immunoreactivity of HDL in the cells of the endocrine part of the pancreas of different animal models was also examined to determine whether the presence of HDL is uniform across different types of rodent species. We also wanted to ascertain if the high-fat diet could affect the subcellular disposition of HDL in islet cells.

## 2. Materials and Methods

### 2.1. Animal Models Utilized in This Study

#### 2.1.1. Groups of Experimental Animal Models

Experimental rodent models were arranged as follows, non-diabetic Wistar control (*n* = 6): STZ-induced diabetic Wistar rats (*n* = 6). A low-fat diet (LFD)-fed GK rat model (*n* = 6), and GK rats fed on an elevated (high)-fat diet (HFD) (*n* = 6), ZL (non-diabetic, Zucker lean: *n* = 6), and ZDF (Zucker diabetic, and fatty: *n* = 6).

##### Wistar Rats

Two-month-old male Wistar rats (250 g, *n* = 12) bred at the Animal Housing Complex, College of Medicine & Health Sciences, United Arab Emirates University (UAEU), were utilized for the experiment. The rodents were laid in polypropylene cages and had access to rodent pellets (Fujairah Feed Factory, Fujairah, United Arab Emirates) and water ad libitum. The Animal House in which they were maintained was kept between 22 °C and 25 °C in a 12/12 h light/dark pattern. This study received the approval of the Animal Research Ethics Committee, UAEU, Al Ain, United Arab Emirates (Approval number: ERA-2022-8481; Approval Date: 9 February 2022).

After acclimatization, the Wistar rats were randomly divided into two groups of 6. One group received an intraperitoneal (i.p.) injection of streptozotocin (STZ, Sigma-Aldrich Chemie GmbH, Taufkirchen, Germany) in citrate buffer (0.5 M, pH 4.5) at a dose of 60 mg/kg of bodyweight. The other group was designated as the normal, non-diabetic group and received the equivalent volume of citrate buffer i.p. In order to confirm diabetes mellitus in the diabetic group, blood drops were taken from the tail vein after an overnight fasting of the rodents and measured with a glucometer (OneTouch^®^ Ultra^®^ 2 Glucometer, LifeScan, Inc., Milpitas, CA, USA). The cut-off blood glucose level of rats in the diabetic group at the start of this study was 126 mg/dL.

##### Goto–Kakizaki Rats

Goto–Kakizaki (GK) murine models were acquired from the vendor (Taconic Inc., Germantown, NY, USA). GK rats are classified as a lean, rodent representative of T2DM. The metabolism of GK (fasting hyperglycemia, insulin resistance, polyuria and vascular complications) is comparable to the human version of T2DM [21]. GK rats were first introduced into the research community by Tohoku University in 1975. A total of six male GK rats were selected and used for immunofluorescence after measuring the total body weight and the blood glucose level. This group of GK rats received regular laboratory rodent pellets and water ad libitum. The animals were kept on this diet for 28 weeks before tissue sample retrieval after decapitation by guillotine.

##### Non-Diabetic Lean, Diabetic and Fatty Zucker Rats

Non-diabetic lean, and diabetic, and fatty Zucker rats were bought from a vendor (Charles River Laboratories, Margate, UK). The Zucker rat was introduced in the 1960s as a rodent model of obesity [22]. The Zucker rats were randomly selected and fed on regular rodent pellets, with water ad libitum, for 28 weeks before sample processing after humane decapitation.

### 2.2. Rodent Representation of Diabetes Mellitus Type 1 (T1DM)

In order to create a rodent imitation of T1DM, Wistar rats were treated with streptozotocin (STZ) injection (60 mg/kg of body-wt) according to a published method [23]. Briefly, STZ was freshly prepared in citrate-buffered solution before injection as described in Section 2. Streptozotocin causes diabetes by binding to the GLUT2 receptor located on the plasma membrane of pancreatic beta cells, after which it alkylates nuclear DNA, resulting in DNA destruction. This DNA lesion results in the stimulation of poly ADP-ribosylation. This process leads to the consumption of ATP/NAD and necrosis of the beta cells of the endocrine pancreas [24].

### 2.3. Effect of Diet on the Cellular Density of HDL

In order to determine whether a fatty diet could influence the cellular density of HDL in pancreatic islet cells, GK rats (T2DM model) were either fed with LFD or HFD as described previously by Howarth et al. [21].

Briefly, 18 male GK rats, aged 8 weeks old, were randomly selected and were either fed on HFD (Catt#: D12492, *n* = 6) or LFD (Cat#: D12450B, *n* = 6). Six other GK rats were fed on a regular rodent diet and served as controls. These rodent diets were purchased from a vendor (Research Diets Inc., New Brunswick, NJ, USA) and kept according to the manufacturer’s instruction. The fat content of the HFD was 34.9 g% compared to 4.3 g% in the LFD. The GK rats were fed on these special diets for a duration of 28 weeks. Water was available to the rodents as desired.

#### Whole-Body Weight, and Glycemic Status

The total body weight and glycemic levels of experimental animals were taken before the retrieval of pancreatic tissue fragments for light and electron microscopy studies. The body weight was measured with Sartorius electronic laboratory balance (Sartorius Lab Instruments GmbH & Co. KG, Goettingen, Germany). A OneTouch-Ultra^®^ 2 Glucometer was utilized to evaluate the level of sugar in the blood taken from the tail vein of the animals [23].

### 2.4. Paraffin Embedding of Tissue Samples

Pancreatic tissues fragments were retrieved expeditiously from the rodents after anesthesia and decapitation, and fixed for 24 h at room temperature in a picric acid-based Zamboni’s fixative, placed in increasing strengths of ethanol, treated with xylol, and later immersed in liquid paraffin wax. The 6 µm-thick sections were made from cured paraffin wax chunks using a Shandon laboratory microtome (Shandon AS325, Kalamazoo, MI, USA), and kept on gelatin-coated glass slides, before staining for HDL according to a previously reported technique [23,25].

### 2.5. Immunohistochemistry of HDL in Islet Cells Using the Avidin–Biotin Complex Staining Method

The 6 µm-thick tissue slices were immersed in xylol and graded ethanol before staining for HDL using the avidin–biotin complex method according to a previous report [23]. In brief, tissue sections were rinsed with phosphate-buffered solution (PBS) prior to the antigen recovery step. The area of the tissue sections was marked with a Dako pen before treatment with hydrogen peroxidase-methanol to neutralize peroxidase endogenous to the tissue section. After a PBS rinse, the pancreatic tissue slices were then treated, at 4 °C, with HDL antibody (Abcam, Waltham, MA, USA, Cat# 34788, dilution: 1:100). After 24 h, the slides of the pancreatic sections were flushed thoroughly in PBS, and immersed in 2° biotinylated antibody (anti-rabbit IgG, dilution: 1:20, Abcam) at 22 °C. The sections were later douched in PBS and treated with 3,3-diamino-benzidine tetrahydrochloride (Sigma-Aldrich), dehydrated and placed in DPX (Dibutylphthalate Polystyrene Xylene) mounting fluid. Photoimages were taken with a digital camera fitted with Axio-Vision version 3.0 (Carl Zeiss, Oberkochen, Germany). The immunohistochemical staining was repeated 4 times.

### 2.6. The Double Labelling Immunofluorescence (IF) Method

In order to ascertain if HDL is located alongside pancreatic hormones in pancreatic islet cells, the double labelling IF method was used based on an established method [26]. Briefly, the sections were treated with xylene, dehydrated in alcohol, and washed in PBS before they were incubated overnight with antibodies against either HDL/insulin or HDL/glucagon at 4 °C. HDL was purchased from Abcam (Waltham, MA, USA) and used at a concentration of 1:100 (Cat# 34788), while insulin (Cat# A0564, dilution 1:500) and glucagon (Cat# A0565, dilution 1:500) were bought from DAKO (Santa Clara, CA, USA). Twenty-four hours after incubation, the pancreatic tissue slices were flushed with PBS before the application of 2° antibodies. The slices were later rinsed in PBS, and covered with CITI Fluore (Science Services GmbH, München, Germany). Photoimages were captured with Zeiss fluorescence microscope (Carl Zeiss). Fluorescence images were merged with a Photoshop Software, version 25.3.1. The double immunofluorescence staining of pancreatic tissue sections was repeated 4 times.

### 2.7. Immunoelectron Microscopy

In order to establish the subcellular location of HDL in the cells of the islets of Langerhans, ultrathin sections were incubated with IgG-conjugated immunogold particles as previously reported by Elabadla et al. [27]. In brief, nickel grids containing ultrathin sections of islet cells were rinsed with water (deionized), incubated in 10% H_2_O_2_ before immersion in 0.01 M PBS (pH 7.3) that contains 0.5 M NH_4_Cl, followed by a quick wash in 1% BSA dissolved in 0.1% Tween-20 solution. The nickel grids were immersed in normal goat serum, before incubation at 4 °C for 24 h in HDL antibodies (Abcam, 1:00 dilution). This step was followed by treatment with GAR (goat-anti-rabbit) IgG molecule that is chemically bonded to 15 nm size gold nano-particles ((TAAB Laboratories Equipment Ltd., Aldermaston, UK, Cat # GEM026-15, dilution at 1:20). The ultrathin sections were later washed in deionized water, and dried on filter paper. The contrasting of the ultrathin sections was performed with C_4_H_6_O_6_U, and C_6_H_8_O_7_Pb dissolved in distilled H_2_O. The grids containing the ultrathin, 40–80 nm slices were inspected using a Philips Tecnai TEM (Transmission electron microscope, Philips, Amsterdam, The Netherlands). Immunoelectron microscopic staining of the grids was repeated 3 times.

### 2.8. Morphometry of HDL, INS- and GLU-Immunoreactive Cells

The total sum of HDL-, INS- and GLU-positive cells in a given pancreatic islet was counted with the use of Image J^®^ version 1.5.4 (NIH, Bethesda, MD, USA) according to a technique published earlier from our laboratory [27]. Six islets per rat per each group were included in the analysis (*n* = 6).

### 2.9. HDL-Containing Beta Cell Granules

The total sum of HDL particles in beta cell granules of GK control rats, and GK rats fed on either a low-fat diet or a high-fat diet was estimated with Image J^®^. IgG-conjugated gold particles per field were counted based on a previously reported technique [27]. Eight electron microscopy fields per rat per group were calculated.

### 2.10. Statistical Evaluation of Data

The data obtained in this study were depicted as the mean ± SEM. Remarkable statistical variations amongst Wistar, GK, Zucker lean and Zucker diabetic fatty sets of rodents were evaluated with Student’s *t*-test. The cut-off for the remarkable (statistical) difference was *p* ≤ 0.05.

## 3. Results

### 3.1. Comparison of Total Body Weight and Glycemic Levels of Rodent Models of Diabetes Mellitus

The weight and glycemic levels of the animal models varied to a wide extent. The mean weight and glycemic levels of control Wistar, STZ-diabetic Wistar (*n* = 6), GK (*n* = 6), ZL, (*n* = 6), and ZDF (*n* = 6) rats are shown in Table 1.

The total body weight of the GK rat model was markedly higher in comparison to that of Wistar models. Normal control Wistar rats had a significantly larger weight compared to their diabetic counterparts. In addition, the mean weight of ZDF rats was remarkably higher than that of age-matched ZL rats. The glycemic level of ZDF rats was distinctly elevated (*p* < 0.007) in comparison to their Zucker lean counterparts. The blood glucose levels of STZ-diabetic Wistar and GK rodents were much higher when compared to non-diabetic Wistar rodents.

### 3.2. Localization of Alpha Lipoprotein (HDL) in Pancreatic Islet Cells

**Figure 1:**
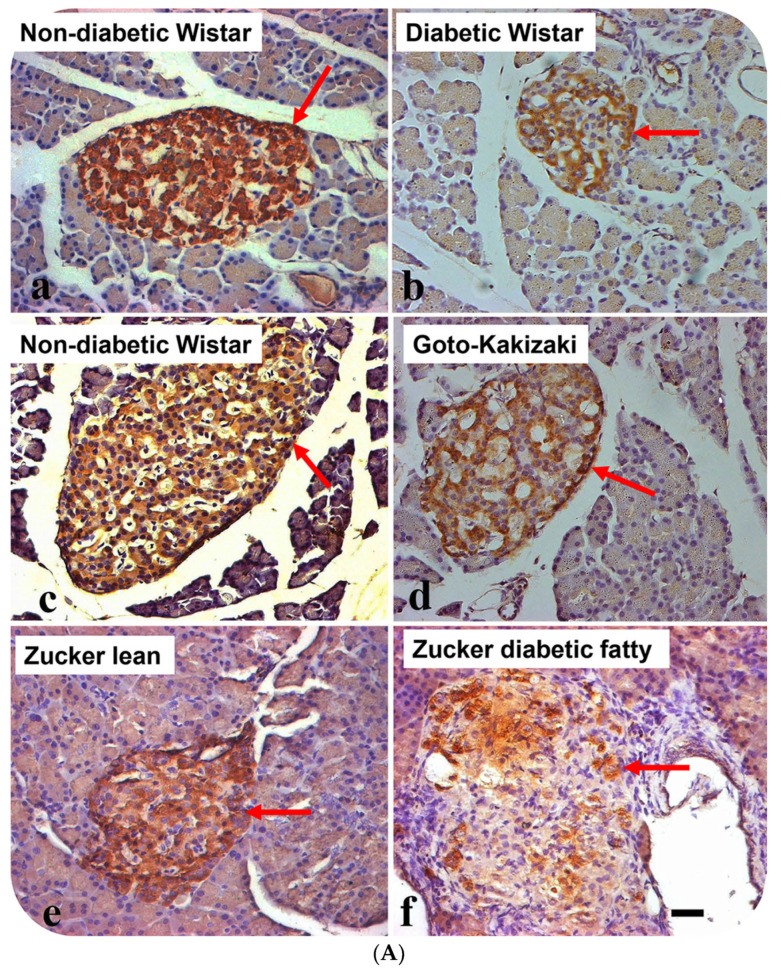

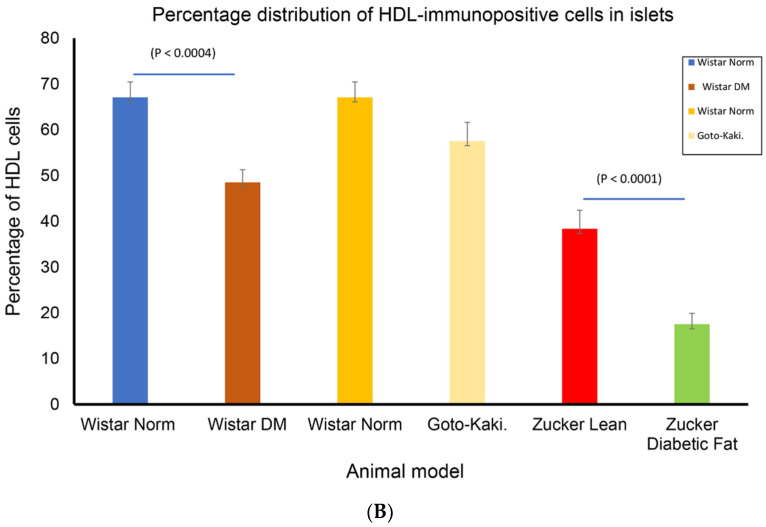
(**A**) Immunohistochemical localization of high-density lipoprotein (HDL) in pancreatic islets of different rat models of diabetes mellitus. (**a**) non-diabetic Wistar, (**b**) diabetic (STZ-treated) Wistar, (**c**) non-diabetic Wistar, (**d**) Goto–Kakizaki (GK), (**e**) Zucker lean, and (**f**) Zucker diabetic fatty. Arrows show islets with HDL-immuno-positive cells. Note that HDL-positive cells are fewer in STZ-diabetic Wistar, GK and Zucker diabetic fatty rats, compared to their respective controls. Data are obtained from six different animals from each set of rats. *n* = 6; linear scale = 50 µm. (**B**) Morphometry of HDL-immunoreactivity in the pancreatic islets of non-diabetic Wistar; STZ-diabetic Wistar; Goto–Kakizaki; Zucker lean, Zucker diabetic fatty rats. Note that the total sum of HDL-immuno-positive cells is markedly (*p* < 0.0004) reduced in STZ-Wistar diabetic, and Zucker diabetic fatty rats when compared to control Wistar rats. The total sum of islet cells that contains HDL was much lower in Zucker diabetic fatty rats compared to other animal models.

The avidin–biotin complex staining method was employed to ascertain whether HDL is located in pancreatic islet cells. Many of the cells resident to the core of pancreatic islets of control Wistar models contain HDL-immunoreactivity. Although, the total sum of HDL-immunoreactive cells was moderately curtailed in GK rats in comparison to control Wistar rats, the calculated difference was not significant. In contrast, the total sum of HDL-containing cells was markedly reduced in Wistar diabetic and Zucker diabetic rats. HDL-containing cells were also significantly lower in pancreatic islet cells of Zucker lean compared to in those of the control Wistar and GK rat models (Figure 1A,B).

### 3.3. Double Labelling Immunofluorescence (IF) of HDL and Islet Hormones

**Figure 2:**
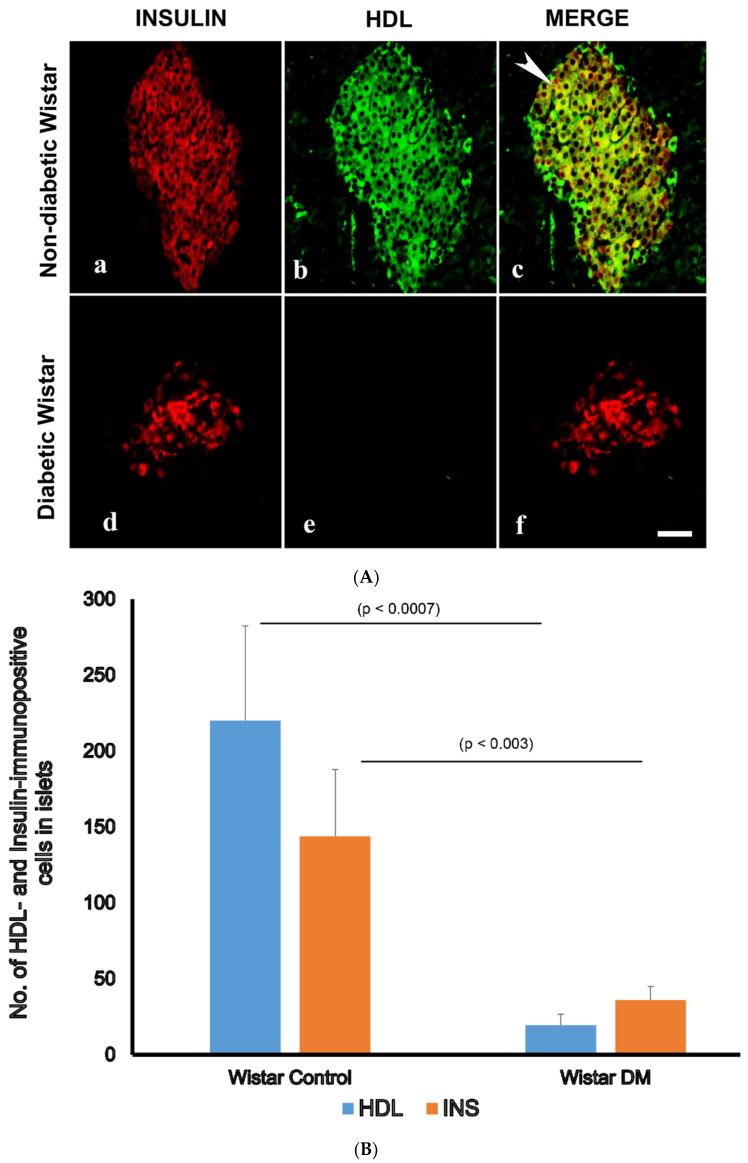
(**A**) Immunofluorescence (IF) images of high-density lipoprotein (HDL)- and insulin (INS)-immunopositive cells in the islets of control (non-diabetic) (**a**–**c**) and diabetic (**d**–**f**) Wistar rats. (**a**) Insulin-positive cells (red) are located in the core of pancreatic islets of normal rats, (**b**) HDL-containing cells (green) are found in central region of control, non-diabetic rats. (**c**) Merged (incorporated) images (red + green = yellow) showing that HDL is co-localized with INS (white arrow heads); (**d**) surviving INS-immuno-positive cells (red) in the pancreatic islet of diabetic rats; (**e**) HDL is not seen in diabetic rat islets; (**f**) merged images of INS (red) and HDL (green) cells have disappeared after the induction of diabetes. *n* = 6; linear scale = 50 µm. (**B**) Morphometry of INS-, and HDL-immunoreactive cells in the islet cells of normal control Wistar and diabetic rats. Note the concomitant and marked (*p* < 0.003) reduction in the number of INS- and HDL-immunoreactive cells after the onset of diabetes mellitus.

**Figure 3:**
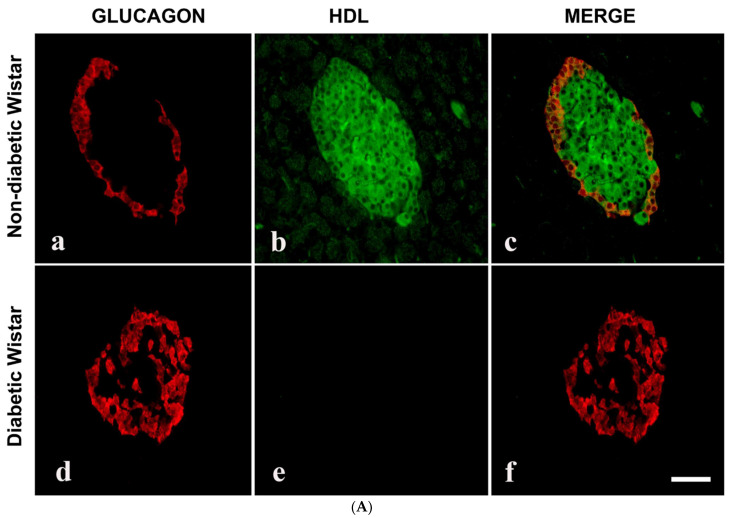

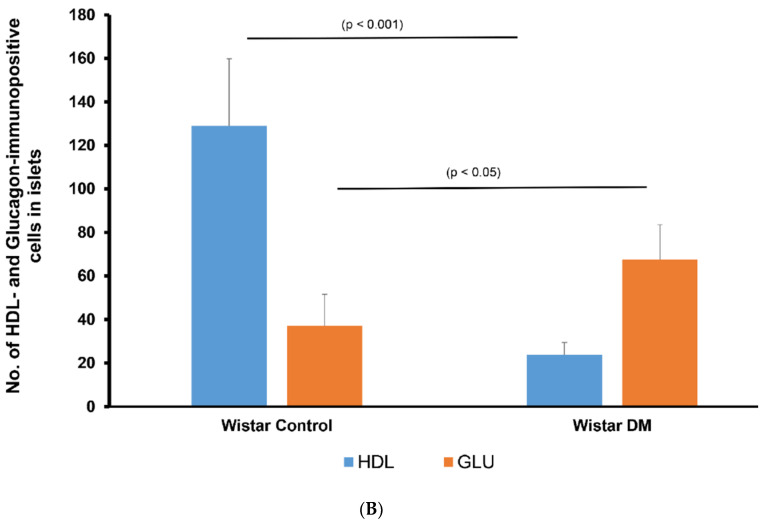
(**A**) Immunofluorescence images of alpha lipoprotein (HDL)- and glucagon (GLU)-immunoreative cells in pancreatic islets of control (non-diabetic) (**a**–**c**) and DM (diabetic) (**d**–**f**) Wistar rats. (**a**) GLU-immunoreactive cells (red) were noted in the rim of islets of Langerhans, whereas (**b**) HDL-containing cells (green) are found mostly in the central region in the non-diabetic rat model. (**c**) Merged image (red + green = yellow) showed that glucagon is not co-localized with HDL; (**d**) GLU-immunoreactive cells (red) in STZ-induced diabetic Wistar rat islets; (**e**) HDL was not observed in diabetic Wistar rat islets; (**f**) merged images of GLU (red) and HDL (green) cells were not seen following the inception of diabetes mellitus. *n* = 6, linear scale = 50 µm. (**B**) Morphometry of the islets of Langerhans of control (non-diabetic) and DM (STZ-induced diabetic) Wistar rats after processing for GLU and HDL immunofluorescence. Note the marked (*p* < 0.001) depletion in the total sum of HDL-immunoreactive cells in the islets and an associated elevation in the total sum of GLU-positive cells.

Since we observed that HDL-containing cells are located in the core of pancreatic islets, we thought that HDL may be present in islet cells that produce pancreatic hormones. The double-labelling IF showed that HDL is located alongside INS in pancreatic islets. In contrast, HDL was not discernable in GLU-producing alpha cells of the pancreas. The IF technique also indicated that the total sum of islet cells containing HDL was markedly (*p* < 0.0007) lower in diabetic rodent models. Concomitantly, the total sum of INS-immunoreactive cells was also remarkably (*p* < 0.003) diminished. In contrast to the reduced number of both HDL- and INS-positive cell in islet cells, the total sum of GLU-immunopositive cells increased in diabetic groups. All of these indicate and support our initial observation that HDL is found alongside INS in the β-cells of the islets (Figure 2A,B and Figure 3A,B).

### 3.4. Transmission Immunoelectron Microscopy of HDL in β-Cells of GK Rats Fed on a High-Fat Diet

**Figure 4:**
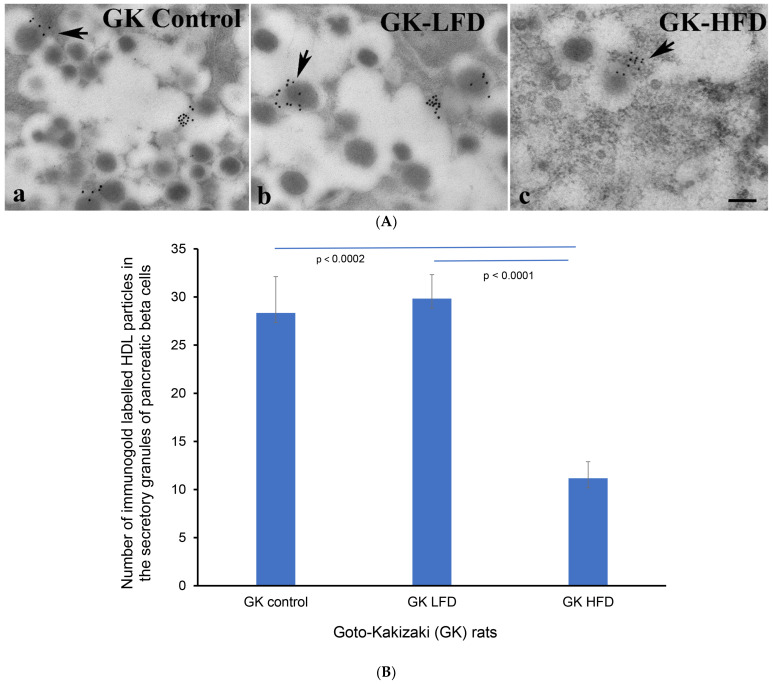
(**A**) Immunoelectron micrographs showing immunogold-labelled high-density lipoprotein (HDL) molecules (black dots at the arrows) in the excretory vesicles of β-cells of (**a**) control GK (fed on regular laboratory chow), (**b**) LFD-GK and (**c**) HFD-GK rats. *n* = 6. GK = Goto–kakizaki; LFD = low-fat rodent chow; HFD = high-fat rodent chow. Scale bar = 5 µm. (**B**) Morphometric evaluation of HDL-coated immunogold particles in the secretory vesicles of β-cells. Note the marked (*p* < 0.0002) reduction in the total sum of HDL-gold-conjugated particles in the secretory vesicles of GK rats fed with HFD.

After showing that HDL is resident to insulin-producing pancreatic beta cells, we conducted a transmission electron microscopic investigation of where HDL is located within the β-cells of the endocrine pancreas of Goto–kakizaki rats that were given either a high-fat diet or a low-fat diet. We showed that immunogold particles conjugated with HDL were localized to the secretory vesicles of β-cells in the pancreas. The number of HDL-conjugated immunogold particles was distinctly (*p* < 0.002) lower than in the GK control rats fed on a regular laboratory chow and LFD GK rats (Figure 4A,B).

## 4. Discussion

The streptozotocin (STZ)-induced diabetic Wistar, GK and Zucker rats are some of the most regularly utilized rodent models for the investigation of the pathophysiology, complications and therapeutic interventions of diabetes mellitus (DM). The hormones of the pancreas contribute to the maintenance of the homeostasis of glucose. The intact regulation of these hormones is therefore paramount to the prevention of diseases of metabolism, such as DM. Hyperglycemia-induced hyperlipidemia is a frequent long-term complication of DM [11] that arises because of altered glucose metabolism and the increased release of lipid products including cholesterol into the blood circulation. Increased blood levels of beta lipoproteins (LDL) and pre-beta lipoproteins (VLDL) contribute to the development of cardiovascular conditions such as atherosclerosis that may eventually lead to the blockade of the arteries that supply the myocardium and eventually a manifestation of myocardial infarction [9]. Patients with a high blood concentration of LDL and with a reduced level of HDL are more prone to developing cardiovascular events [28,29,30]. It has also been shown that individuals with a low level physical activity tend to have a low blood level of HDL [31].

The disparity in the weight of the different rodent models used in this study is not surprising, since they are raised from different genetic backgrounds. However, it is obvious from the results that the more obese the subject, the greater the likelihood of developing hyperglycemia. It has been shown that obesity contributes to the initiation and triggering of DM [32]. Several studies have shown that Wistar rats fed on a high-fat diet for a prolonged period develop obesity, dyslipidemia and diabetes [33]. In another study, GK rats fed on HFD for four weeks developed diabetic thoracic aortic lesion [34]. However, it is also possible to find a non-obese, diabetic person. Non-obese diabetics have been reported in several countries [35,36,37]. The glycemic levels of GK and Zucker (lean and diabetic) rats were markedly elevated when compared to those of the control rats. This shows that irrespective of the type of diabetes, hyperglycemia is a key and noxious sign of DM [2,38]. Hyperglycemia is the main trigger of OS, which stimulates a cascade of molecular events that leads to dyslipidemia, micro- and macrovascular complications associated with DM [39,40,41,42,43,44].

We demonstrated the presence of HDL in the islet cells of the pancreas with immunohistochemistry, immunofluorescence and transmission electron microscopy. Further investigation showed that HDL was indeed located in pancreatic beta cells. This is not surprising, because a large number of bioactive molecules have been localized to the endocrine pancreas. Indeed, in one study, lipoprotein receptors have been localized to the plasma membrane of pancreatic beta cells. These receptors were able to translocate lipoprotein molecules into the internal compartment of the cell [45]. The study also showed that, in contrast to the effect of LDL and VLDL, HDL prevents the release and invigoration of proapoptotic molecules in the beta cells of the pancreas. In addition, several peptides, including but not limited to apelin, pleiotrophin, ghrelin and urocortin have also been detected in the beta cells of the pancreas [27,46,47,48].

We showed that the total sum of HDL-positive cells was markedly reduced in DM, with a simultaneous lowering of the total sum of INS-producing pancreatic beta cells. This is clear evidence that HDL and insulin do indeed co-localize to the same islet cell. The co-localization of HDL with INS in β-cells may point to the modulation of the activity of beta cells by HDL. In our investigation, we also used transmission electron microscopy to identify the exact cytoplasmic organelle of the β-cells of the islets that contains HDL. This shows that HDL and insulin are packaged together into the same secretory granules where they might be released together into circulation by exocytosis.

What could be the task of HDL in the pancreas? HDL is reported to prevent the apoptotic decay of pancreatic beta cell [45]. Several other studies have also shown that HDL can prevent the apoptotic degeneration of the mitochondria in pancreatic beta cells [49]. Others have reported that HDL can stimulate insulin secretion from beta cell lines [50,51]. The mechanism by which HDL stimulates insulin from beta cells is not well understood, nevertheless, many reports have suggested that this may be through several mechanisms such as the inhibition of sphingosine 1-phosphate (S1P), increased Pdx1 expression, mitigation of the stress experienced by the endoplasmic reticulum (ER), and stimulation of the phosphorylation of Ak-strain transforming (Akt), 5′ AMP-polypeptide kinase (AMP-K), and extra-cellular signal-transduction-regulated kinase½ (Erk½) [52,53]. It has also been proposed that HDL binds to the ATP-binding cassette pump or transporter (ABCA-1) protein and its subfamily G counterpart (ABCG-1) to initiate insulin release via exocytosis [54].

How does HDL fit into the concept of insulin metabolism and secretion? What has already been reported about insulin anabolism reports that insulin biosynthesis is controlled at two major points (transcriptional, and translational) [55]. The genes that influence this aspect of insulin metabolism include but are not limited to homeobox-1 of the pancreas and duodenum (PDX-1) [56], MafA [57], forkhead box protein A2 [58], and homeobox protein Nkx2 [59]. Following the translational phase of insulin biosynthesis, the proinsulin molecule is further matured in the rough endoplasmic reticulum before moving into the Golgi apparatus where small vesicles of immature insulin are formed [60]. The mature insulin and C-peptide molecules are later packed in secretory vesicles in association with peptides such as amyloid polypeptide [61,62]. Our results suggest that HDL co-localizes with insulin and could therefore participate in the regulation of insulin function.

The secretion of insulin is triggered by glucose, starting with the attachment of glucose to the GLUT2 receptor on the plasma membrane of beta cells. This process stimulates, glucokinase, protein kinase C, and calcium ions, which in turn facilitate the development of exocytosis in β-cells [63,64]. The extent of glucose-induced insulin release may be affected by circulating nutrients such as lipid molecules [65] and some amino acid [66] molecules, melatonin [67], and GLP-1 [20,68]. The insulin thus released by exocytosis passes into the fenestrated capillaries to circulate in both the system and pulmonary blood circuits.

## 5. Conclusions

This study shows that alpha lipoprotein (HDL) is present in the beta cells of the endocrine pancreas, where it colocalizes with insulin. Transmission immuno-electron microscopy revealed that HDL is found in the excretory vesicles of pancreatic β-cells. The total sum of islet cells that embodies HDL molecules was significantly lower in all animal models of diabetes. These results indicate that HDL may contribute to the regulation of insulin metabolism.

## Figures and Tables

**Table 1:** Weight and glycemic levels of different rodent models of diabetes mellitus.

No	Rodent Model	Total wt. (g)	NF Blood Glucose Concentration (mg/dL)
1	Wistar (normal) rats	310.8 ± 25.2 #	38.0 ± 1.7 *
2	Wistar (diabetic, STZ-treated) rats	241.7 ± 44.1	174.0 ± 3.6
3	Goto–Kakizaki (GK) rats	391.2 ± 28.1 ##	124 ± 19.7 **
4	Zucker lean (ZL) rats	5 04.33 ± 63.36	117.8 ± 5.1
5	Zucker diabetic (ZDF) rats	738.5 ± 33.9 ♦♦♦	133.7 ± 7.5 ***

* # *p* < 0.007 vs. Wistar diabetic; ** ## *p* < 0.0007 vs. Wistar control; ♦♦♦ *** *p* < 0.008 vs. Zucker lean. wt = weight; NF = non-fasting; *n* = 6 for all groups.

## Data Availability

All data supporting the observations of this study are available within this article.
